# An Inhibitory Role of the G-Protein Regulator AGS3 in mTOR-Dependent Macroautophagy

**DOI:** 10.1371/journal.pone.0008877

**Published:** 2010-01-26

**Authors:** Benjamin Groves, Hilde Abrahamsen, Heather Clingan, Michael Frantz, Lauren Mavor, Jeffrey Bailey, Dzwokai Ma

**Affiliations:** 1 Department of Molecular, Cellular, and Developmental Biology, University of California Santa Barbara, Santa Barbara, California, United States of America; 2 Neuroscience Research Institute, University of California Santa Barbara, Santa Barbara, California, United States of America; 3 Department of Biochemistry, Institute for Cancer Research, The Norwegian Radium Hospital, Oslo University Hospital, Montebello, Oslo, Norway; 4 Centre for Cancer Biomedicine, Faculty of Medicine, University of Oslo, Montebello, Oslo, Norway; Roswell Park Cancer Institute, United States of America

## Abstract

Macroautophagy is a cellular process whereby the cell sequesters and recycles cytosolic constituents in a lysosome-dependent manner. It has also been implicated in a number of disorders, including cancer and neurodegeneration. Although a previous report that AGS3 over-expression promotes macroautophagy suggests a stimulatory role of AGS3 in this process, we have found that knock-down of AGS3, unexpectedly, also induces macroautophagy, indicating an inhibitory function of endogenous AGS3 in macroautophagy. Interestingly, AGS3 phosphorylation is decreased upon induction of mammalian target of rapamycin (mTOR)-dependent macroautophagy. Moreover, unlike wild-type AGS3, over-expression of an AGS3 mutant lacking this modification fails to enhance macroautophagic activity. These observations imply that AGS3 phosphorylation may participate in the modulation of macroautophagy.

## Introduction

In eukaryotes, macroautophagy (hereafter simply referred to as autophagy) is a universal metabolic process whereby the cell encloses portions of its cytoplasm in a double membrane-bound structure called an autophagic vacuole or autophagosome. The autophagomes subsequently fuse with lysosomes, leading to the degradation and recycling of the sequestered content [1]. While autophagy was originally viewed as an inducible cellular mechanism to provide an energy source during short-term starvation, it has subsequently been shown that constitutive autophagy mediates the elimination of protein aggregates or damaged organelles and thus plays a protective role in multiple cell types [2], [3]. On the other hand, high levels of autophagy can lead to cell death [4]. These observations suggest that autophagic activity needs to be closely monitored and regulated within a cell. Consistent with this notion, de-regulation of autophagic function has been proposed to participate in neurodegenerative disease and cancer [5], the innate immune response [6], as well as aging [7]. Although genetic screens have identified many proteins encoding essential autophagic components, the characterization of the mechanisms by which a cell regulates autophagic activity remains incomplete. One major pathway that modulates autophagy involves the mammalian Target of Rapamycin (mTOR), a Ser/Thr kinase that functions as a signaling hub to control cell proliferation, growth, and survival by regulating transcription, translation, as well as autophagic activity [8]. Activation and inhibition of mTOR, respectively, repress and enhance autophagy, most likely by controlling the assembly of ATG1 complex during the induction of autophagy [9], [10], [11]. Although mTOR represents a major regulator of autophagy, several mTOR-independent pathways of autophagy have been found [12], [13].

Activator of G-protein Signaling 3 (AGS3; also known as GPSM1) was first identified during a functional screen for mammalian proteins that activate heterotrimeric G-protein signaling in a receptor-independent manner in *Saccharomyces cerevisiae* [14]. Sequence analysis of AGS3 reveals a 70 kDa protein with a three-module structure, including seven tetratricopeptide repeats (the TPR domains) at the N-terminus [15], [16], a linker domain in the middle, and four G-protein regulatory motifs (the GPR or Goloco domains) at the C-termnius [17], [18]. Biochemical approaches have demonstrated that the GPR domains of AGS3 preferentially bind and stabilize GDP-bound Gαi subunits [19], [20], [21], [22]. By acting as a guanine dissociation inhibitor (GDI) of the Gαi subunit, AGS3 may promote the dissociation of Gαβγ or inhibit the re-association of Gαi with the Gβγ dimer. As a consequence, AGS3 could potentially inhibit the Gαi-dependent pathways but enhance Gβγ-regulated signaling. Because of its abundant expression in neurons, most AGS3 studies have focused on the brain. Studies in rodents have indicated that the AGS3 level is up-regulated in specific brain regions, the prefrontal cortex (PFC) and nucleus accumbens (NAC), during the late withdrawal from drug or alcohol administration and that this up-regulation plays a necessary and active role in various addiction-associated processes, including behavioral sensitization and the reinstatement of drug-seeking behavior [23], [24], [25]. AGS3 also functions in asymmetric cell fate by controlling mitotic spindle orientation during brain development [26]. Despite the importance of AGS3 for brain function, we and others have shown that AGS3 is present in multiple tissues and cell types [14], [20], [22], [27], [28], [29], [30] and displays a limited co-localization with the markers of ER and Golgi [28]. Consistent with these observations, our previous study revealed a potential role of AGS3 in the structure and/or function of the Golgi apparatus [30]. More recently, the study of AGS3 null mice has indicated an unexpected function of AGS3 protein in metabolic and cardiovascular function [31], suggesting that a major function of AGS3 *in vivo* lies in cell metabolism.

As a major AGS3-interacting protein [14], [19], [21], [22], [32], Gα_i3_ has been shown to control autophagic sequestration in colon carcinoma cells [33]. Given the interaction between AGS3 and Gα_i3,_ Pattingre *et al.* have over-expressed AGS3 and its truncation mutants in human colon cancer HT-29 cells and examined their effects on autophagy [28]. These authors show that AGS3 over-expression stimulates autophagy, whereas over-expression of either the TPR or the GPR domain suppresses it. However, whether and how endogenous AGS3 regulates autophagy is unknown. Moreover, considering that mTOR is a major regulator of autophagy, it should be interesting to explore the relationship between AGS3 and the mTOR pathway in the context of autophagy. The answers to the above questions will provide insight into the mechanism by which the intersection of autophagy and G-protein signaling is regulated.

## Materials and Methods

### Cell Culture and Transfection

COS7 [30], HEK293 (ATCC CRL-1573) or HeLa [30] cells were cultured in Advanced D-MEM medium (GIBCO) supplemented with 4% fetal bovine serum, 2 mM glutamine and 1X penicillin-streptomycin (Cellgro). Cell starvation was performed by incubating cells in HANKs or EBSS medium supplemented with 25 mM HEPES for 2 hrs. With the exception of the GFP-LC3 assays, cells were transfected with DNA (0.4 µg/ml) using FuGENE6 HD (Roche) or siRNA (20 nM) using Lipofectamine RNAiMAX (Invitrogen). Cell lysates were collected 24 hrs or 48 hrs after transfection, respectively, for DNA or RNA transfections. The following siRNAs targeting human AGS3 (GPSM1) were purchased from QiagenAGS3 siRNA1: GGG CGC UGG AAU ACC ACA A; AGS3 siRNA2: CCG AGU UCU ACG AGA GGA A.

### DNA Constructs and Site-Directed Mutagenesis

Rat AGS3 cDNA was obtained as previously described [30]. HA-AGS3, HA-AGS3TPR (residues 1–472) as well as the HA-ASG3GPR (residues 461–650) constructs were PCR amplified from the wild-type AGS3 construct and cloned into the pcDNA3 vector containing an N-terminal HA-epitope. The full length AGS3 GPR ser/thr mutant was made using the Quikchange Lightning Site-Directed Mutagenesis kit (Stratagene) and verified by DNA sequencing. Serines (S) and threonines (T) present within the last 183 amino acids of AGS3 were mutated to alanine, this includes S467, S468, S478, S482, S483, T503, T507, S516, T518, S520, T523, S532, S533, S535, S544, S547, T554, S583, S584, T602, S610, S630, S636, and S650.

### Cell Lysis

Cells were lysed in ice-cold RIPA lysis buffer (50 mM Tris HCl [pH 80.], 150 mM NaCl, 1 mM EDTA, 1% Triton X-100, 0.1% SDS and 1% sodium deoxycholate). Directly before addition of the lysis buffer, Complete protease inhibitors (Roche) and 1 mM PMSF were added. Following the clearing of lysates by centrifugation for 10 min at 4°C, the lysates were adjusted to the same volume and concentration of total protein as determined by a Non-Interfering Total Protein assay (G Biosciences). Lysates were incubated for 15 min at 90°C in SDS-PAGE loading buffer supplemented with β-mercaptoethanol. Samples were stored at −20°C.

### Antibodies

The following commercially available antibodies were used in this study: rabbit polyclonal LC3B antibody (Cell Signaling), rabbit polyclonal HA.11 antibody (Covance), mouse monoclonal actin antibody (BD Biosciences), goat polyclonal DyLight 680 anti-rabbit IgG (Jackson ImmunoResearch) and goat polyclonal DyLight 680 anti-mouse IgG (Jackson ImmunoResearch). We also raised a rabbit polyclonal antibody directed against a GST-fusion of the C-terminal region (a.a. 461–650) of AGS3 (characterized below).

### Immunoprecipitation

Cells were lysed as described above, with the exception that cells were solublized in ice-cold NP-40 lysis buffer (50 mM Tris-HCl [pH 8.0], 150 mM NaCl, 1 mM EDTA and 1% NP-40). After protein quantification, lysates were incubated with the appropriate primary antibody at 4°C for 2 hrs followed by incubation with protein G-Sepharose (Invitrogen) for anther 2 hrs at 4°C. The sepharose beads were washed four times in ice cold lysis buffer and the bound proteins were eluted with SDS-PAGE sample buffer supplemented with β-mercaptoethanol at 90°C for 15 min.

### SDS-PAGE and Western Blot Analysis

Samples were separated by SDS-PAGE electrophoresis (6%–13% gradient gels, BioRad), and transferred to Immobilon 0.45 µm PVDF membranes (Milipore) using a Semi-Dry Electroblotting System (Owl). Membranes were incubated with the appropriate primary antibody overnight at 4°C in a 1∶1 mixture of Odyssey blocking buffer and PBS supplemented with 0.1% Tween-20. Excess primary antibody was removed by performing three 10 min washes with PBS supplemented with 0.1% Tween-20. Secondary antibody incubation was performed in a 1∶1 mixture of Odyssey Blocking buffer and PBS supplemented with 0.1% Tween-20 and 0.02% SDS for 30 min, following which the membranes were washed with PBS supplemented with 0.1% Tween-20 (three times for 5 min each) followed with PBS for 1 min and dried in the dark. Quantification was performed using an Odyssey Infrared Imaging System.

### GFP-LC3 Assay

One day before siRNA transfection, 8X10^6^ HEK GFP-LC3 cells (a gift from Sharon Tooze; Cancer Research UK) were plated in 60 mm dishes. Transfection with siRNA was performed using 10 µl Lipofectamine RNAiMAX transfection reagent (Invitrogen) and 50 nM siRNA. 48 hrs after transfection the cells from each plate were detached using Trypsin/EDTA and replated onto 4 fibronectin coated coverslips (for microscopy) as well as reseeded onto a fresh 60 mm dish (for lysis and verification of knock-down by western blot). The following day, cells growing on coverslips were treated either with regular growth media (DMEM supplemented with 10% FBS), regular growth media containing 100 nM bafilomycin A, starvation media (EBSS) or starvation media (EBSS) with bafilomycin A1 for 2 hrs. Thereafter, cells were washed twice in cold PBS and placed on ice where they were permeabilized for 5 min in ice-cold 0.05% saponin containing PEM buffer (80 mM PIPES pH 6.8, 5 mM EGTA and 1 mM MgCl_2_) prior to 15 min fixation in 4% paraformaldehyde. Coverslips were mounted onto slides in Moviol supplemented with 2 µg/ml Höechst and analyzed for GFP-LC3II puncta formation on the high throughput Olympus ScanR microscope using the fully automated acquisition and analysis platforms. For each coverslip 64 images were acquired using the 40X objective and the number of GFP-LC3II puncta was analyzed for each cell. On average 1500–3000 cells were identified per coverslip.

### Real-Time PCR

Total RNA from HEK293 cells treated with a non-targeting control siRNA or AGS3 siRNA1 were isolated using an RNeasy Plus Mini Kit (QIAGEN) and quantified with a nanodrop spectrophotometer (Thermo Scientific). cDNA was synthesized by reverse-transcription using the RevertAid First-Strand cDNA synthesis kit (Fermentas) with a nonspecific oligo(dT) primer and real-time PCR was performed in triplicate for each sample using the iCycler iQ Real-Time PCR System (Bio-Rad). Approximately 100 bp of the cDNA of choice was amplified using two independent sets of primers targeting distinct areas of the LC3 mRNA. The results from these primers were normalized using those from four sets of primers targeting the cDNA sequences of several “house-keeping” gene products (GAPDH, GPI (3′), HMBS and HPRT1).

## Results

### Both Knock-Down and Over-Expression of AGS3 Increase LC3II Levels

**Figure 1:**
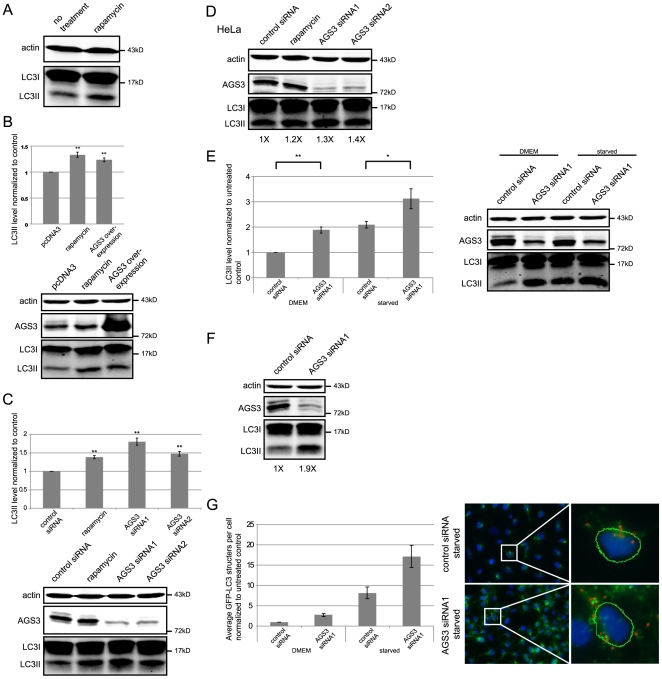
Both knock-down and over-expression of AGS3 lead to an increased LC3II level in HEK293 and HeLa cells. Cells transfected with DNA (0.4 µg/ml) or siRNA (20 nM) were lysed 24 or 48 hrs after transfection, repectively, unless otherwise noted. β-actin was used as a loading control. (A) The effect of rapamycin (100 nM, 4 hrs) on the level of LC3II in HEK293 cells. (B) The impact of AGS3 over-expression on LC3II in HEK293 cells. Cells transfected with an empty vector (pcDNA3) were used as a negative control. (C) The influence of AGS3 knock-down on LC3II in HEK293 cells. Cells transfected with a non-targeting siRNA were used as a negative control. (D) The influence of AGS3 knock-down on LC3II in HeLa cells. Western blot LC3II band intensities are normalized to that of the control siRNA treated cells (arbitrarily set as 1X). (E) HEK293 cells were treated with either a non-targeting control siRNA or AGS3 siRNA1 and either starved (2 hrs) or not. (F) GFP-LC3 stable HEK293 cells were treated with either a non-targeting control siRNA or AGS3 siRNA1 and endogenous LC3 was assayed by western blot. Western blot band intensities are normalized to that of the control siRNA treated cells (arbitrarily set as 1X). (G) HEK293 cells stably expressing GFP-LC3 were treated with AGS3 siRNA1 (50 nM, 72 hrs) and either starved (2 hrs) or not; a higher AGS3 knock-down efficiency was obtained for the HEK293 GFP-LC3 stable cells when using a higher siRNA concentration and a longer incubation time. This could be due to differences in cell origin. The number of GFP positive puncta structures were quantified as described in the “Materials and Methods”. For the sake of clarity normalized GFP-LC3II counts/cell (n = 6, approximately 10,000 cells/condition) are shown. Two statistical tests (ANOVA and single-sample Student's t-test) performed on LOG transformed data revealed that the AGS3 effect was significant across all experimental conditions (p<0.05 for Starved, p<0.01 for all others). The images on the left side are representative full size acquired images, the inset to the right shows a close-up of one cell with indication of detected GPF puncta (red circles). The green dotted lines enclose the cell nuclei. Representative blots and images are shown (AGS3 was detected using a rabbit polyclonal antibody characterized below). Data presented in bar graphs is the average result from at least three independent experiments except in (G). Error bars: standard error of the mean. Asterisks - * = p<0.05, ** = p<0.01, paired Student's t-test.

Upon induction of autophagy, a cytosolic protein, microtubule-associated protein 1 light chain 3 (LC3I) is processed and covalently linked to a phosphatidylethanolamine moiety to yield LC3II. This lipid-modified LC3II associates specifically with autophagic membranes [34], and thus its levels can be used as to measure of the amount of autophagic compartments [35]. To assure ourselves that the change in LC3II levels can be used as an indicator of altered autophagic activity in HEK293 cells, we treated cells with rapamycin (100nM, 4 hrs) and measured both the total LC3I and the LC3II levels by quantitative western blot analysis. Rapamycin induces autophagy by forming a complex with FKBP12 and directly inhibiting the mTOR activity [36], [37], [38], [39]. Indeed, the ratio of the levels of LC3II to LC3I as well as those of LC3II alone were increased by approximately 40% and 35%, respectively, under our experimental conditions (Figure 1A). Due to several caveats associated with LC3I detection such as its sensitivity to degradation in SDS-sample buffer and to freezing-thawing [35], the total LC3II level relative to actin has been shown to be a more reliable indicator of autophagy than the ratio of LC3II to LC3I. Thus, we measured the total LC3II level and used actin as a loading control in the remainder of the study.

We next investigated the effects of over-expression and knock-down of AGS3 on the levels of LC3II in HEK293 cells. Consistent with a previous report by Pattingre, et al [28], the over-expression of AGS3, but not an empty vector (pcDNA3) resulted in a 24% increase in LC3II levels (Figure 1B). Interestingly, compared to a non-targeting control siRNA, LC3II levels also rose in cells treated with either of two siRNAs targeting different sites of AGS3 mRNA (by 80% for siRNA1 and 48% for siRNA2; Figure 1C). Similar results were obtained in HeLa cells (Figure 1D). Since both siRNAs exert a similar effect, we used siRNA1 for subsequent studies. To ask whether AGS3 also impacts induced autophagy, we compared the effect of depleting AGS3 on the LC3II levels under normal growth or starved conditions; the latter of which is thought to trigger autophagy by indirectly inhibiting mTOR activation via a less well understood signal transduction pathway [40]. As shown in Figure 1E, suppression of AGS3 further increased the LC3II level in starved cells.

Although an increased LC3II level, when measured by western blot, is generally indicative of an increase in autophagosome number, the relationship is not always linear [35]. As an independent assay of autophagosome formation we utilized an HEK293 cell line stably expressing a GFP fusion of LC3 [41], [42] and measured the influence of knocking-down AGS3 on the formation of GFP positive structures under normal growth or starved conditions. As with endogenous LC3I and LC3II proteins, GFP-LC3I is not associated with a membrane and is thus found in the cytoplasm, whereas GFP-LC3II exhibits a punctate pattern due to its association with the autophagosome membrane. Since the assay was performed after cell permeabilization (see “Materials and Methods”), the free GFP-LC3I is able to diffuse out, while the membrane-bound GFP-LC3II is retained. Thus, the number of GFP-positive puncta correlates with the number of autophagic structures. Consistent with the results obtained above using western blot analyses (Figure 1C-E), as well those from the quantification of endogenous LC3II in the GFP-LC3 cell line (Figure 1F), the fluorescent images show that AGS3-depleted cells exhibit much more green puncta compared to the control cells under both normal growth (data not shown) and starved conditions, a conclusion which was further confirmed by the quantification (Figure 1G). Relative to the control cells, a 3- and 2.2-fold increase of the number of GFP-LC3II puncta was observed in AGS3 knock-down cells in normal growth and starvation media, respectively. It should be noted that the ratio of the non-starved AGS3 siRNA1 treated sample to the starved control sample in the GFP-LC3II analysis is not the same as in the western blot analyses; this could be due to the use of different cell lines (HEK293 vs. HEK293 GFP-LC3 stable line), or as a result of the differing natures of the two assays (the LC3II protein level vs. the number of GFP-LC3II puncta structures). Taken together, the above data suggest that loss of AGS3 leads to an increased number of autophagosomes within the cell under both normal growth and starved conditions.

### AGS3 Knock-Down Increases Autophagic Activation

**Figure 2:**
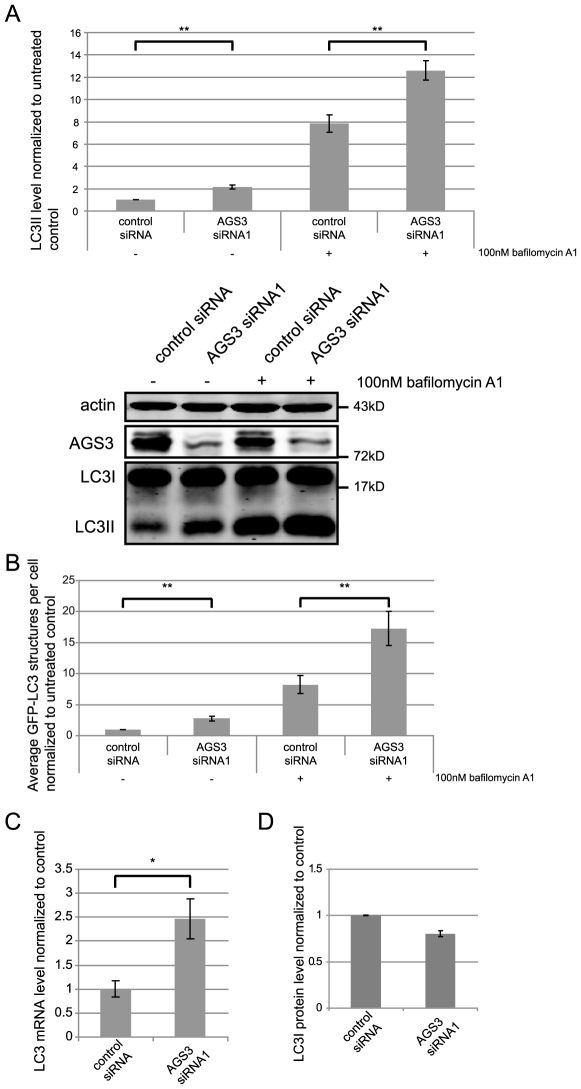
The effect of AGS3 depletion on LC3II persists when the fusion between autophagosomes and lysosomes is inhibited in HEK293. siRNA-mediated knock-down of AGS3 was performed as described in Figure 1. (A) The impact of AGS3 knock-down on the LC3II level in the presence of bafilomycin A1. Cells transfected with a non-targeting control siRNA or AGS3 siRNA1, were treated with either DMSO alone (as a negative control) or bafilomycin A1 (100 nM, 2 hrs). (B) The influence of AGS3 suppression on the number of GFP-LC3II puncta in HEK293 stable cells treated with bafilomycin A1 (100 nM, 2 hrs). Cells stably expressing GFP-LC3 were first transfected with a non-targeting control siRNA or AGS3 siRNA1 and the GFP-LC3II puncta was quantified and the statistical analysis performed as described in Figure 1; in both conditions the difference between control siRNA and AGS3 siRNA1 samples is statistically significant (p<0.01). Normalized GFP-LC3II counts/cell (n = 4, approximately 10,000 cells/condition) are shown. (C) The effect of AGS3 depletion on the mRNA level of LC3. HEK293 cells were treated with a non-targeting control siRNA or AGS3 siRNA1; total RNA was collected, reverse-transcribed and analyzed by real-time PCR. Results were normalized to four “house-keeping” gene products. (D) The LC3I levels in HEK293 cells treated with either a non-targeting control siRNA or AGS3 siRNA1 determined by western blot. Data presented in all bar graphs is the average result from three (A, C & D) or four (B) independent experiments. The values of untreated control and knock-down cells presented in (B) are provided for the sake of comparison and are the same as those that appear in Figure 1G. Error bars: standard error of the mean. Asterisks - * = p<0.05, ** = p<0.01, paired Student's t-test. Representative blots are shown. Similar results as those presented in 2A were obtained in HeLa cells (data not shown).

The number of autophagosomes found within a cell depends on both the induction of autophagy, as well the rate of their degradation, which occurs upon fusion with the lysosome. AGS3 knock-down could conceivably increase the number of autophagosomes and thus the level of LC3II by either inducing autophagosome formation or by preventing their turnover. It is important to distinguish between these two scenarios since they indicate opposite effects of AGS3 in autophagy (stimulation in the former and inhibition in the latter). To address this question, we made use of the same two approaches described above to investigate the impact of AGS3 depletion on the bafilomycin A1-induced increase in autophagosome number. As a vacuolar-type H^+^-ATPase inhibitor, bafilomycin A1 blocks the fusion between autophagosomes and lysosomes and thus the degradation of LC3II [43], [44]. If AGS3 knock-down inhibited the fusion between autophagosomes and lysosomes, we would expect that the level of LC3II or the number of GFP-LC3II puncta in AGS3 knock-down cells would not increase any further in the presence of bafilomycin A1. However, as shown in Figure 2, the increase of both the level of LC3II (Figure 2A) as well as the number of GFP-LC3II puncta (Figure 2B) persisted in AGS3 knock-down cells treated with bafilomycin A1 (100 nM, 2 hrs), suggesting that AGS3 does not function to inhibit the fusion between autophagosomes and lysosomes. A similar observation was made when we used two inhibitors of lysosomal enzymes, pepstatin A and E64d (data not shown). When combined with the data from Figure 1, these results indicate that AGS3 knock-down increases autophagosome number by promoting their formation rather than by inhibiting their degradation.

Further support for the above model comes from comparing the effects of AGS3 depletion on the mRNA and protein levels of LC3. Although a real-time PCR analysis revealed an ∼2.5 fold increase in the level of LC3 mRNA in AGS3 knock-down cells compared to the control (Figure 2C), indicating elevated synthesis of LC3 protein, there was no corresponding increase of the level of LC3I protein (Figure 2D). These observations suggest that the amount of LC3I converted to LC3II is increased in cells treated with AGS3 siRNA.

### AGS3 Phosphorylation Within Its GPR Domain Is Sensitive to the Induction of mTOR-Dependent Macroautophagy

**Figure 3:**
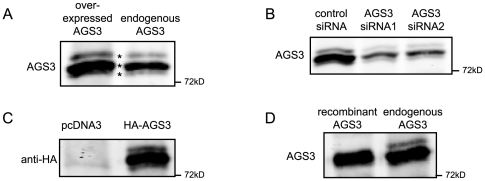
A rabbit polyclonal antibody detects an AGS3 post-translational modification in HEK293 and HeLa cells. DNA and siRNA transfections were performed as described in Figure 1. (A) In HeLa cells, exogenously over-expressed untagged-AGS3 as well as endogenous AGS3 exhibited three differently migrating bands (indicated by asterisks). (B) Characterization of the putative AGS3 bands using lysates collected from HeLa cells transfected with AGS3 siRNAs. The presence of three differently migrating bands which are sensitive to the AGS3 siRNAs is also evident in HEK293 cells (Figures 1C, 1E & 2A). (C) Analysis of the band pattern of an HA-tagged AGS3 expressed in HeLa cells. (D) Comparison of the mobility between recombinant AGS3 expressed in *E. coli* and endogenous AGS3 from HeLa cell lysate.

To further characterize the role of endogenous AGS3 in autophagy, we raised a polyclonal rabbit antibody against the GPR domain of AGS3. Interestingly, our antibody recognized three differentially migrating species in HeLa cells (Figures 1D & 3), as well as in HEK293 (Figures 1 & 2) and COS7 (see below). We chose to investigate these bands in HeLa because compared to HEK293 and COS7, HeLa cells tend to express exogenously introduced protein at a lower level, thus facilitating the separation and analysis of the triplet on SDS-PAGE.

To determine whether the triplet visualized by western blot was indeed AGS3, we knocked-down endogenous AGS3 in HeLa cells with either of two siRNAs targeting AGS3 (Figure 3B). Indeed, all three bands were significantly reduced following siRNA transfection, indicating that they are derived from AGS3. Moreover, exogenous expression of an HA-tagged AGS3 resulted in a similar triplet on SDS-PAGE (Figure 3C), suggesting that these bands are generated via covalent modification(s) and/or protein degradation. Finally, the comparison between the mobility of endogenous AGS3 and that of a recombinant AGS3 (Figure 3D) further shows that the slowest-migrating band of the triplet is most likely covalently modified.

**Figure 4:**
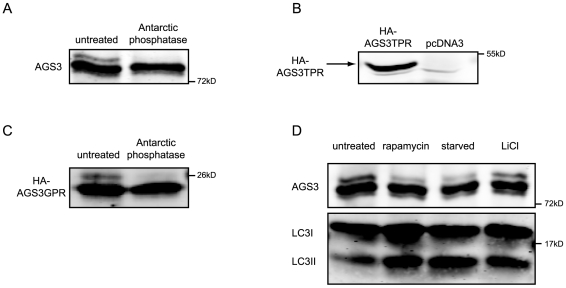
Characterization of the slowest-migrating species of AGS3 and its sensitivity to mTOR-dependent autophagy. DNA transfections were performed as described in Figure 1. (A) Western blot analysis of AGS3 immunoprecipitated from HeLa cell lysates in the presence or absence of Antarctic phophatase. (B) Western blot analysis of a truncated AGS3 construct (HA-AGS3TPR; a.a. 1–472) consisting of the N-terminal TPR domains immunoprecipitated from HeLa lysates. The lower light band likely represents a non-specific signal as it appears in both lanes. (C) Western blot analysis of the phosphatase treatment of a truncated AGS3 construct (HA-AGS3GPR; a.a. 461–650) consisting of the C-terminal GPR domains immunoprecipitated from HeLa lysates. (D) The influence of mTOR-dependent and –independent autophagic inductions on the triplet of AGS3. Autophagy was induced in HeLa cells by inhibiting mTOR activity, via either rapamycin (100nM, 4 hrs) or starvation (2 hrs), or by an mTOR-independent pathway via LiCl (10 mM, 24 hrs).

Early studies have shown that AGS3 can exist in a phosphorylated state ([45], [46]; Phosphosite.org). To test if the slowest-migrating band represents a phosphorylated form of AGS3, we immunoprecipitated endogenous AGS3 using our AGS3 antibody, incubated the immunoprecipitated AGS3 in the presence of Antarctic phosphatase (Figure 4A) or Calf Intestine Phosphatase (data not shown), and analyzed AGS3 migration on SDS-PAGE by western blotting. As evident from Figure 4A, the slowest-migrating species of AGS3 is susceptible to the phosphatase treatment, implying that it is a phosphorylated species. The same result was obtained using an HA-tagged AGS3 immunoprecipitated with a rabbit polyclonal antibody against the HA-epitope (data not shown). As it has been reported that the GPR or GoLoco domain of AGS3 can be phosphorylated [45], we next determined whether the phosphorylation that we observed also occurs in the same domain. For this, we made use of two constructs; one construct containing an HA epitope fused to the GPR domain (residues 461–650; HA-AGS3GPR) and the other consisted of an HA epitope fused to the TPR domain and part of the linker region (residues 1–472; HA-AGS3TPR). Although HA-AGS3TPR migrates as one band (Figure 4B), HA-AGS3GPR migrates as two species (Figure 4C). Moreover, the level of the slower migrating species of HA-AGS3GPR was greatly reduced after the phosphatase treatment (Figure 4C). Collectively, our data show that the slowest-migrating band represents a species of AGS3 which is phosphorylated in its GPR domain.

Since our above study implies a role of AGS3 in the formation of autophagosomes, we examined whether the level or phosphorylation of AGS3 is altered upon autophagic induction. Although rapamycin (100 nM, 4 hrs) or starvation (Hanks' medium, 2 hrs) treatment did not significantly change the total AGS3 protein levels, both treatments caused a great decrease in the level of the slowest-migrating form of AGS3 (Figure 4D). We also assessed whether the level of the slowest-migrating band was also sensitive to the addition of LiCl (10 mM, 24 hrs) to the culture medium. In contrast to rapamycin and amino acid starvation, LiCl activates autophagy independent of mTOR [12], [47]. Figure 4D shows that whereas LiCl treatment increased the level of LC3II, it failed to impact the level of the slowest-migrating AGS3 band. Taken together, our data indicate that a pool of AGS3 is phosphorylated in the GPR domain and that this modification is sensitive to the induction of mTOR-dependent autophagy. Since the knock-down of AGS3 does not have a detectable impact on the phosphorylation of S6 kinase 1, an established target of mTOR ([48], [49]; data not shown), AGS3 likely functions to modulate autophagy downstream of mTOR.

### The AGS3 GPR Phospho-Mutant Fails to Induce Autophagy When Over-Expressed

**Figure 5:**
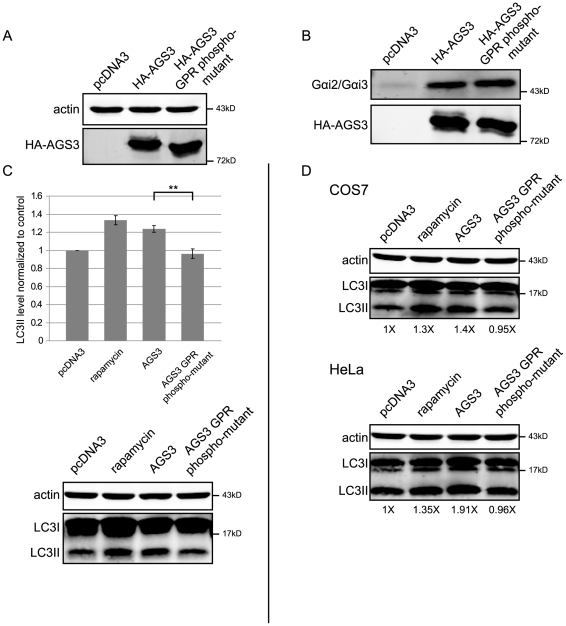
Over-expression of the AGS3 phospho-mutant does not induce autophagy in HEK293 HeLa or COS7 cells. DNA transfections were performed as described in Figure 1. (A) Western blot analysis of the AGS3 GPR phospho-mutant (all Ser and Thr within last the C-terminal 183 a.a. mutated to Ala) expressed in HEK293 cells. (B) Co-immunoprecipitation of Gαi2/Gαi3 with either wild-type AGS3 or the AGS3 phospho-mutant from HEK293. (C) Assessment of the ability of AGS3 GPR phospho-mutant to induce autophagy in HEK293 cells. HEK293 cells transfected with the vector alone, AGS3 or AGS3 GPR phosphor-mutant were lysed and LC3II levels was assayed. (D) Assessment of the ability of AGS3 GPR phospho-mutant to induce autophagy in COS7 and HeLa cells. Representative blots are shown. Data presented in the bar graph in (B) are the average results from at least three independent experiments. Error bars: standard error of the mean. Asterisks = p<0.01, paired Student's t-test. Western blot band intensities in (D) are shown as normalized to that of vector (i.e. pcDNA3) transfected cells (arbitrarily set as 1X).

The above finding prompted us to explore whether the GPR-phosphorylated species of AGS3 plays a part in the AGS3 autophagy phenotype. To address this question, using the full-length wild-type AGS3 as a template, we systematically mutated all 22 of the serine and threonine residues within the GPR domain (a.a. 469–650), as well as 2 serines lying just outside, into alanines (tyrosine is not present within the GPR domain) by site-directed mutagenesis. As expected, this AGS3 mutant does not display the band corresponding to the slowest-migrating species of AGS3 (Figure 5A). The following two observations imply that the loss of phosphorylation of this mutant is not due to a global folding defect. First, the AGS3 GPR phospho-mutant was expressed at a comparable level with that of wild-type AGS3 (Figure 5A). Second, the mutant retained the ability to co-immunoprecipitate Gα_i2_/Gα_i3_ (Figure 5B). Importantly, unlike wild-type AGS3, over-expression of the AGS3 phospho-mutant had no influence on the LC3II level in HEK293 cells (Figure 5C). Similar results were found in COS7 and HeLa cell lines (Figure 5D). These observations raise the possibility that the phosphorylation of AGS3 within its GPR domain may play a role in regulating mTOR-dependent autophagy.

## Discussion

While a previous study has shown that AGS3 over-expression increases autophagic activity in human intestinal HT-29 cells, suggesting a stimulatory role of this protein in autophagy [28], it has remained unknown whether or how endogenous AGS3 functions in autophagy. Here, we provide evidence that, similar to AGS3 over-expression, AGS3 knock-down also enhances autophagic activity in HEK293 and HeLa cells. This conclusion, albeit somewhat unexpected, is based on the following two sets of observations. First, AGS3 knockdown leads to an increased steady-state accumulation of LC3II protein level (as measured by western blot), as well as an increased number of autophagic structures (as measured by GFP-LC3II puncta) (Figure 1). Second, when the lysosomal degradation of LC3II is blocked by bafilomycin A1 in AGS3 knock-down cells, a further increase in the level of LC3II protein or in the number of GFP puncta is observed, indicating that AGS3 depletion likely enhances the formation of autophagosomes (Figure 2). It is intriguing to note that the AGS3 knock-out mice exhibit a lean phenotype [31], in light of recent research suggesting that autophagy regulates lipid metabolism [50].

Our finding that both over-expression and knock-down of AGS3 cause increased autophagic activity seems peculiar but is consistent with other scenarios where over-expression of a wild-type protein results in a dominant negative effect [51], [52]. One explanation for our observation is that AGS3 normally functions in the context of a complex composed of multiple components in a specific stoichiometry. Under these conditions, either an elevated (in the case of over-expression) or decreased (in the case of knock-down) level of AGS3 could inhibit the formation of this functional complex, and thus cause a similar phenotype. As further support for this model, we previously reported that both over-expression and knock-down of AGS3 results in a similar dispersal of several trans-Golgi network (TGN) proteins [30]. In this scenario, one may also imagine that a more moderate increase (instead of the relatively high level that occurs when AGS3 is over-expressed) of AGS3 level could actually inhibit autophagy. Identification of additional AGS3-interacting proteins should provide clues on this topic.

The observations that the phosphorylation level of AGS3 is inversely correlated with the autophagic activity (Figures 4), and that the over-expression of a GPR phospho-mutant of AGS3 fails to induce autophagy as wild-type AGS3 does (Figure 5) raise the intriguing possibility that a phosphorylation switch controls the function of AGS3 in autophagy. The fact that the level of the slowest-migrating phosphorylated band of AGS3 is greatly decreased when autophagy is induced by rapamycin or starvation, but not by LiCl, implies that ASG3 is acting along the mTOR-dependent autophagic pathway. How does AGS3 inhibit autophagy? Based on our data, one attractive hypothesis is that mTOR or one of its downstream effectors phosphorylates AGS3 and the phosphorylated AGS3 contributes to the mTOR-mediated suppression of autophagy. When mTOR activity is inhibited by either rapamycin or starvation, AGS3 becomes de-phosphorylated and the autophagic activity is increased accordingly. In a similar fashion, ULK1-Atg13-FIP200 regulation of autophagic activity is mediated by mTOR association and phosphorylation [10], [11]. Previously, an AGS3 phosphorylation site at Ser 630 was identified in a phosphoproteomic analysis [46]. Unfortunately, AGS3 constructs that we characterized carrying mutations of the site to Ala or Asp did not affect the level or mobility of slowest-migrating band, implying that another phosphorylation site or sites are involved in our observations reported here (data not shown). Moreover, although LKB1 has been reported to phosphorylate the GPR domain of AGS3 [45], LKB1 depletion did not exert a detectable impact on the slowest-migrating band under our experimental conditions (unpublished observation). Identification of the kinase and phosphatase controlling the formation and removal of the slowest-migrating AGS3 phosphorylation band, as well as the corresponding site(s) of phosphorylation, would certainly provide further insight. An alternative possibility is that AGS3 may inhibit autophagy independent of its phosphorylation status and the disappearance of the slowest-migrating phosphorylated band of AGS3 is merely a consequence of autophagic degradation. Under this condition, the inability of the phospho-mutant of AGS3 to induce autophagy can be due to the loss of a yet-to-be identified autophagy factor, which normally modulates autophagy by binding to the AGS3 GPR domain irrespective to the phosphorylation status of AGS3, but can no longer recognize the mutated AGS3 form. In this aspect, identification of additional AGS3-interacting proteins may prove helpful. Finally, we would also like to point out that, while the GPR phospho-mutant of AGS3 co-immunoprecipitates Gα_i2_/Gα_i3_ to a level comparable to wild-type AGS3, our data do not rule out a role of Gα_i3_ in autophagy.

Another related question is the precise role of AGS3 in autophagy. Both the previous [28] and our current studies have indicated that AGS3 functions during an early stage of autophagy, instead of the fusion step between autophagosomes and lysosomes. One possible, albeit less likely, mode of AGS3 action is through the transcriptional control of autophagy regulating genes, as revealed by our result showing that AGS3 knock-down increases the mRNA level of LC3 (Figure 2C). Another mechanism by which AGS3 may affect the induction of autophagy is by modulating autophagosome formation in the cytosol. In this regard, the elevated LC3 mRNA observed in cells depleted of AGS3 may be a consequence of AGS3-mediated induction of autophagy. It is noteworthy that increasing evidence has suggested a potential link between the ER/Golgi compartments and the autophagic machinery [53], [54]. Moreover, as a major AGS3 interacting partner, Gα_i3_ is enriched at the Golgi apparatus in many cell types [55], [56], [57], [58], [59], [60]. Considering that a pool of AGS3 resides at the ER/Golgi compartments [28] and that knock-down of AGS3 leads to an altered distribution of several TGN proteins [30], one intriguing question to be addressed is whether AGS3 affects autophagosome formation via its effect on these vesicular compartments.
